# Out of sight out of mind? A life cycle-based environmental assessment of goods traded by the European Union

**DOI:** 10.1016/j.jclepro.2019.118954

**Published:** 2020-02-10

**Authors:** Sara Corrado, Tomas Rydberg, Felipe Oliveira, Alessandro Cerutti, Serenella Sala

**Affiliations:** a European Commission, Joint Research Centre, Via Enrico Fermi 2749, I-21027, Ispra, VA, Italy; b IVL Swedish Environmental Research Institute, 100 31, Stockholm, Sweden

**Keywords:** Trade, Environmental impacts, Life cycle assessment, Embodied emissions, Sustainable development goal 12, Sustainable production and consumption

## Abstract

Ensuring responsible production and consumption is one of the United Nations’ Sustainable Development Goals (SDGs) to which the European Union (EU) has committed. An increasing body of literature has demonstrated that global trade flows are key contributors to the environmental impacts of consumption. Indeed, very often developed countries import fuels and other resources from developing ones, displacing a large share of environmental burdens related to consumption of goods outside their boundaries. This paper has a triple goal. Firstly, it assesses the environmental impacts of traded goods with a bottom-up approach, adopting life cycle assessment (LCA) and identifying hotspots related to EU consumption. Secondly, it analyses the extent to which the trade of goods is contributing to the environmental impacts of EU apparent consumption. Finally, it compares the contribution of environmental impact of EU traded goods against overall global impacts. Forty representative products imported or exported by the EU were selected based on their relevance in mass and economic value according to official trade statistics. LCA was applied to these products using the EU Environmental Footprint method. The results were then upscaled in order to be representative of the entire impact of traded goods in the EU. Overall, consumption in the EU resulted to cause considerable environmental impacts outside EU boundaries and impacts of imports and exports were mostly associated with few products groups, which either were traded in large quantities (e.g. “Fuels and mineral oils”) or had a high impact intensity compared to the others (e.g. “Pulp of wood and other cellulosic material” for land use).

## Highlights

• Method for assessing the environmental impact of traded goods based on Life Cycle Assessment.
• Relatively few product groups account for a large share of environmental impacts.
• The environmental impacts of import is higher than export for almost all the impact categories.
• Environmental impacts of import is dominated by “Fuels and mineral oils”.
• Environmental impact of export is dominated by “Iron and steel”, “Machineries”, and “Vehicles”.

## Graphical abstract

**Figure:**
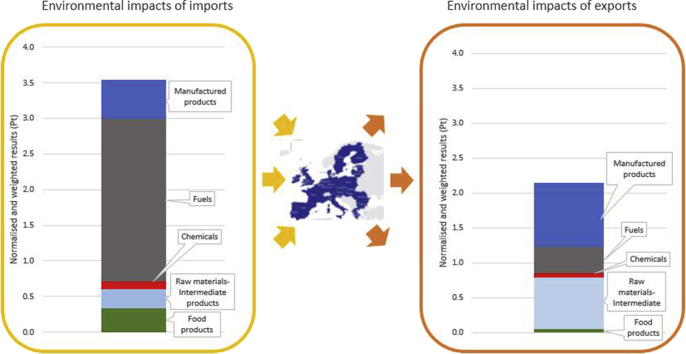

## 1 Introduction

Addressing the environmental sustainability of production and consumption is a key challenge on the global political agenda. Several initiatives and policies aimed to reduce environmental impacts urge a radical transformation of production and consumption patterns (EEA, 2017).

By adopting the 10 Year Framework of Programmes on Sustainable Consumption and Production Patterns at the United Nations Conference on Sustainable Development Rio+20 in 2012 (United Nations (UN), 2012), the global leaders acknowledged the importance of taking action and changing ways in which we produce and consume, in order to achieve global sustainable development. This was also highlighted in the UN Sustainable Development Goal (SDG) 12 (UN, 2015), aimed to ensure sustainable and responsible consumption and production patterns. The European Commission (EC) committed to fully integrate the SDGs in the EU policy framework and in EU priorities, by assessing the current status and identifying the most relevant sustainability concerns (European Commission (EC), 2016). The progress towards sustainable consumption and production patterns does not solely concern EU domestic activities, since consumption is a driver of several activities happening outside the EU borders. The Communication “Trade for all” (EC, 2015) supports the transition towards a more responsible trade and investment policy, whereas the seventh Environment Action Plan (7th EAP) (EC, 2013) highlights the need to reduce environmental impacts caused by EU consumption beyond the EU’s borders.

Evidences from scientific literature underlined that, in a globalised economy, where raw materials, semi-finished and finished goods are largely traded, the growing demand of products within developed countries generates considerable pressures on the environment and causes severe impacts, partly occurring outside the area where products are used or consumed. Indeed, between 10% and 70% of the global environmental and social impacts happen outside the area of consumption (Wiedmann and Lenzen, 2018) and in the period from 1995 to 2011, increasing pressures on greenhouse gas emissions, energy and material use, and blue water consumption were displaced through the trade of goods (Wood et al., 2018). In addition, EU consumption is responsible for about 10% of the global deforestation embodied in goods, almost entirely taking place outside the EU (Cuypers et al., 2013), contributing to global warming and biodiversity loss. In relation to this, four EU countries, namely Germany, France, United Kingdom, and Italy, were among the six world countries causing the highest share of biodiversity loss outside their national boundaries after the United States and Japan (Lenzen et al., 2012).

These figures are of particular interest in light of the increasing global population and the related expected growing consumption, as well as of the environmental degradation we are facing. Therefore, when assessing the sustainability of consumption and production patterns, impacts embedded in imported and exported products and related ethical issues cannot be neglected.

All abovementioned studies calculated environmental impacts of consumption displaced through the trade of goods by means of environmentally extended input-output tables (EEIOT), either multi-region or single-region, or by combining EEIOT and process-based life cycle assessment (LCA), leading to the so-called hybrid EEIOT – LCA (Malik et al., 2018).

Up to the authors’ knowledge, the present study is the first attempt to assess the environmental impacts of traded products entirely relying on process-based LCA. Process-based LCA and EEIOTs have both pros and cons. Process-based LCA has the advantage of being very often more detailed than EEIOTs as it allows for high granularity and flexibility. On the other hand, EEIOTs have the advantage of capturing well macro-scale overall figures and connections between economic sectors. However, it is most likely that EEIOTs cover less environmental interventions, impact categories and elementary flows, namely flows entering and leaving the system under analysis, such as resources and environmental emissions, than process-based LCA, leading to a lower coverage of key environmental impact categories (Beylot et al., 2019). Moreover, process-based LCA allows to model ecoinnovation scenarios more easily by acting on specific product-related features along the entire product life cycle.

This study aims to analyse the environmental impacts caused by goods traded by the EU in the timeframe 2000–2014, through an approach based on process-based LCA of representative products. The study has three objectives: i) to identify the environmental hotspots associated with goods traded by the EU, both in terms of products, emissions, and use of resources; ii) to analyse the extent to which the environmental impacts associated with goods traded by the EU are contributing to the overall impacts of EU apparent consumption; iii) to assess how goods traded by the EU contribute to global environmental impacts.

## 2 Materials and methods

The assessment of the environmental impacts of traded goods by the EU was conducted performing process-based LCAs of a selection of representative products upscaled to cover the entire range of imported and exported products. The procedure to assess the environmental impacts of imports and exports is detailed in the following sections.

### 2.1 Selection of representative products and leading trade countries

The selection of product groups, representative products, and, in the case of imports, of representative countries of origin was based on the mass and the economic value of traded products reported in EU official trade statistics, namely the COMEXT database (Eurostat, 2018). In COMEXT database, product groups are classified following the Harmonized Commodity Description and Coding System (HS) (UN, 2017) with two digits (HS2), and the Combined Nomenclature (CN) with 8 digits (CN8) (EC, 1987). Four years in the timeframe 2000–2014 were considered, i.e. 2000, 2005, 2010, and 2014. The selection of products was done for the year 2010, and the same representative products were considered for 2000, 2005, and 2014.

For both imports and exports, fifteen HS2 product groups were selected, representing the largest imported and exported product groups by mass. In addition, five product groups were identified based on their economic importance, i.e. the most important products traded in economic value terms (excluding them if already included in the fifteen product groups selected by mass). For each of the resulting twenty HS2 product groups, a representative product was selected following the CN8 nomenclature, based on having the largest share of the imports or exports by mass within that HS2 group. The so chosen representative products are listed in Table 1.

**Table 1:** List of product groups and representative products selected for imports and exports. Only the amount of imports and exports for selected product groups are reported.

		Import		Export		
Category	Product group	Representative product	Imported amount in 2010 (100 kg)	Representative product	Exported amount in 2010 (100 kg)	Import/export in 2010
Food products	08-Fruit and nuts	Bananas	1.25E+08			
	10-Cereals	Maize	9.78E+07	Wheat	2.86E+08	34%
	12-Oilseeds	Soybeans	1.79E+08			
	15-Animal or vegetable fats	Crude palm oil	1.02E+08			
	23-Food residues	Oilcake	3.08E+08			
Raw materials-Intermediate products	25-Lime, cements and other materials	Broken or crushed stone	6.14E+08	Portland cement	4.03E+08	152%
	26-Ores, slag and ash	Non-agglomerated iron ores and concentrates	1.50E+09	Agglomerated iron ores and concentrates	1.22E+08	1230%
	39-Plastics	Polyethylene […] in primary forms	1.18E+08	Polyethylene […] in primary forms	2.02E+08	58%
	44-Wood and products	Birch in the rough	2.97E+08	Spruce or silver fir “Abies alba Mill.”, sawn or chipped	2.03E+08	146%
	47-Pulp of wood or other cellulosic material	Semi-bleached or bleached non-coniferous wood pulp	1.03E+08	Semi-bleached or bleached coniferous wood pulp	1.26E+08	82%
	72-Iron and steel	Semi-finished products of iron or non-alloy steel	3.63E+08	Bars and rods, of iron or non-alloy steel	4.84E+08	75%
Fuels	27-Fuels and mineral oils	Crude oil	1.07E+10	Motor spirit	1.62E+09	660%
Chemicals	28-Inorganic chemicals	Anhydrous ammonia	1.45E+08	Sulphuric acid	1.24E+08	117%
	29-Organic chemicals	Methanol	1.81E+08	Acyclic ethers	1.13E+08	160%
	31-Fertilisers	Urea	1.35E+08	Ammonium sulphate	1.17E+08	115%
Manufactured products	30-Pharmaceuticals			Products for therapeutic or prophylactic purposes	7.53E+06	
	48-Paper and products			Paper and paperboard	2.00E+08	
	71-Precious materials	Imitation jewellery	9.63E+05	Coin	3.79E+05	254%
	73-Articles of iron or steel			Line pipe	1.15E+08	
	84-Machineries	Parts suitable for use solely or principally with compression-ignition internal combustion piston engine	9.88E+07	Parts of machinery	1.54E+08	64%
	85-Electrical equipment	Photovoltaic cells	8.40E+07	Electrodes of graphite or other carbon	5.45E+07	154%
	87-Veichles	Cars and other motor vehicles	6.69E+07	Motors caravans	1.45E+08	46%
	88-Aircrafts			Aeroplanes and other powered aircrafts	7.61E+05	
	90-Precision instruments	Mechano-therapy appliances	7.25E+06	Instruments and appliances used in medical, surgical or veterinary sciences	5.47E+06	133%

To model the country of origin of the representative products imported into the EU, it was decided to consider the three countries with the highest exporting mass for each of the CN8 representative products. The extent to which representative products cover the respective product group and representative countries cover the import of the same product from all the countries is reported in Supplementary materials.

### 2.2 Life cycle assessment

The environmental impact of imported and exported representative products was calculated through LCA, using the software GaBi (Thinkstep AG, 2018). The system boundaries were set from cradle to gate, namely starting from the production of materials, up to the point of the production chain where the traded representative products are imported or exported. This implies that in the cases where exported products are produced with materials imported from extra-EU countries, the impacts of raw materials production are accounted both under imports and exports. The impacts were calculated with the life cycle impact assessment method of the Environmental Footprint (EF) (EC, 2013) in the version 1.8 (EC, 2017) , excluding the contribution of long-term emissions. The EF method allows calculating the impacts for 16 distinct impact categories, namely impacts due to: climate change, acidification, ozone depletion, eutrophication (terrestrial, marine, and freshwater), photochemical ozone formation, particulate matter, ionising radiation, freshwater ecotoxicity, human toxicity (cancer and non-cancer), land use, water use, resource use (mineral and metal, and fossil). For some of the impact categories (i.e. acidification, terrestrial eutrophication, land use, and water use) regionalised characterisation factors were available. However, life cycle inventory (LCI) datasets selected to model the representative products did not report regionalised elementary flows, therefore average global characterisation factors were applied.

Within this paper, results of LCA are expressed in two different ways, allowing different types of considerations. In most of the sub-sections, the results are reported as characterised results per each impact category. Moreover, in sub-section 3.2.1, results are expressed using a single score, calculated by applying normalisation and weighting. Normalisation was performed using the global normalisation factors updated from Sala et al. (2017), whereas the weighting adopted the set published by Sala et al. (2018) (both normalisation and weighting factors are reported in Supplementary materials).

The production phase of each representative product was modelled choosing data reflecting as much as possible the actual production method. When available, datasets from the Gabi LCA Database (Thinkstep AG, 2018) or ecoinvent v2.2 database (Frischknecht et al., 2005) were used. If no suitable dataset was found in those databases, the production was modelled with a combination of data from LCA reports, and other technical literature. Overall, due to lack of specific data, the selection of the most appropriate data was based on expert judgement. A selection process to identify the data that could be used as best proxy was needed, for example, when data representative of the technologies in place were missing, when specific data coming from each of the importing countries were not available, or when more than one source was providing data. Negative emissions of heavy metals to agricultural soil reported in some of the ecoinvent datasets for agricultural products were revised considering the emissions factors reported in the Agrifootprint database (Blonk Blonk Consultants, 2015). These changes were done because such negative emissions might be due to uncertainties in the modelling rather than a real uptake of heavy metals from the soil (Koch et al., 2015).

Transport of imported products from producing countries was modelled according to the following general rules. Transport of goods from production site to the port or airport in Extra EU countries was made on a case–by-case basis, considering the specific characteristics of the location of production. The share of goods imported from extra-EU countries by sea and/or plane was retrieved from Eurostat statistics (Eurostat, 2018). Means of transport by sea from receiving port to the capital city were modelled based on Eurostat’s transport database (Eurostat, 2018). Shipment by airplane was modelled as being delivered directly to capital cities in the EU.

Transport of goods by rail and road from production sites situated in Extra EU countries to capital cities in Intra EU countries was modelled based on Eurostat data. The share between means of transport was based on the potential mass transported by each mean.

### 2.3 Upscaling of representative products to the total amount of traded products

The LCA results obtained for the representative products were upscaled in order to estimate the environmental impacts of all traded goods.

**Fig. 1:**
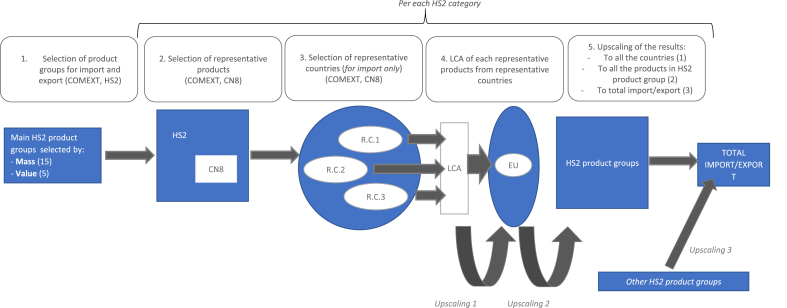
Representation of the selection and upscaling procedure for product groups, representative products, and, in the case of imports, representative countries. R.C. representative countries.

Three types of upscaling were performed, all based on the proportion between mass of products. (Fig. 1). The first upscale, done for imports only, consisted in scaling the mass of each representative product imported from representative countries to the amount imported from all the countries of origin, as described in section 2.1 (Upscaling 1, Fig. 1). Then, the representative products were upscaled to the product groups (Upscaling 2, Fig. 1). Thirdly, the impacts of selected product groups were upscaled to the total imported and exported products (Upscaling 3, Fig. 1).

### 2.4 Assessment of the relevance of impacts of EU traded products at EU and global scales

In order to explore the extent to which traded goods are contributing to the overall impacts of consumption in the EU, the environmental impacts of imports and exports were compared first with the ones generated domestically in the EU as calculated by Sala et al. (2019) following the methodology developed by Sala et al. (2015), and secondly with the impacts of EU apparent consumption. The impacts of EU apparent consumption, which differs from real consumption because it does not consider changes in stock levels between years, were calculated as: impact of domestic activities+ impact of imports – impact of exports. In this study, the apparent consumption does not reflect the final consumption because the analysis of trade flows is not only focused on final products, but includes also categories of products that are further transformed in the EU or elsewhere. For example, imported crude oil could be refined in the EU and then used for industrial purposes.

Furthermore, to investigate to which extent EU consumption is contributing to overall global impacts through trade, the impacts of EU traded products were compared with global impacts. Global normalisation factors developed for LCA were used as a reference for the global impacts. Since global normalisation factors are constantly updated, the most recent version of global normalisation factors available was considered, based on an update of the set of normalisation factors published by Sala et al. (2017), who quantified the environmental impacts generated globally according to the 16 EF impact categories. The list of global normalisation factors is reported in Supplementary materials.

## 3 Results and discussion

This section reports results related to: i) the selection of the representative products; ii) the environmental impact assessment of the products traded by the EU complemented with information on the relative share of impact due to product groups and to specific substances, i.e. emissions into the environment and resources used along the life cycle of products; and iii) the contribution of traded goods respectively to the environmental impacts of EU apparent consumption, and to global impacts. All the results are presented for the year 2010.

### 3.1 Selected representative products

The list of product groups and respective representative products selected for the analysis is reported in Table 1, both for imports and exports. For simplicity, the product groups and the representative products are referred to with an abbreviated nomenclature. The extended names from the official classification systems (HS2 and CN8) are reported in Supplementary materials. The product groups were clustered in five categories, according to their nature: “Food products”, “Raw materials-Intermediate products”, “Chemicals”, “Fuels”, and “Manufactured products” (Table 1). The selected HS2 product groups corresponded to respectively 93% and 70% of the total imported products in 2010 by mass and value, and to 80% and 76% of the exported products, according to the same criteria. The proportion between the selected product groups and the total amount of imported and exported goods was very similar for the other years analysed, meaning that the assumption of considering the same product group for different years allowed covering a considerable share of imported and exported products in terms of both mass and value.

Sixteen of the analysed product groups were relevant both in terms of imports and exports. However, except for “Plastics”, the representative products selected within the product groups were different. On average, for the same product group, representative products selected for imports were more basic products, whereas for exports they were higher value-added products. For example, for the product group “Iron and steel” the representative product “Semi-finished products of iron or non-alloy steel” was selected for imports, while “Bars and rods, of iron or non-alloy steel” was selected as representative product for exports. This tendency reflects the fact that, in terms of mass, the EU is mainly importing raw materials and intermediate products and exporting a larger amount of manufactured products.

### 3.2 LCA results

An overview of the environmental impacts of products traded by the EU for the analysed years is reported in Table 2. The ratio between impact of imports and exports for each year and each impact category is reported in the Supplementary materials.

**Table 2:** Environmental impacts of products traded by the EU.

Impact category	Acronym	Unit	2000		2005		2010		2014	
			Import	Export	Import	Export	Import	Export	Import	Export
Climate change	CC	kg CO_2_ eq	8.45E+11	4.86E+11	1.09E+12	6.12E+11	1.02E+12	6.96E+11	1.08E+12	7.83E+11
Ozone depletion	ODP	kg CFC-11 eq	7.01E+05	2.10E+04	8.45E+05	2.75E+04	8.29E+05	3.06E+04	7.76E+05	3.49E+04
Acidification	AC	molc H^+^ eq	1.51E+10	2.74E+09	1.88E+10	3.52E+09	1.78E+10	4.02E+09	1.90E+10	4.62E+09
Photochemical ozone formation	POF	kg NMVOC eq	6.87E+09	1.53E+09	8.45E+09	1.99E+09	7.99E+09	2.27E+09	8.19E+09	2.55E+09
Eutrophication, marine	MEU	kg N eq	2.17E+09	8.05E+08	2.59E+09	8.06E+08	2.45E+09	1.04E+09	2.64E+09	1.27E+09
Eutrophication, terrestrial	TEU	molc of N eq	1.91E+10	5.00E+09	2.32E+10	6.03E+09	2.18E+10	7.10E+09	2.27E+10	8.18E+09
Eutrophication, freshwater	FEU	kg P eq	6.09E+07	6.25E+07	1.18E+08	8.60E+07	8.02E+07	9.43E+07	9.68E+07	1.10E+08
Particulate matter	PM	disease incidences	2.14E+05	6.08E+04	2.66E+05	8.02E+04	2.42E+05	9.06E+04	2.61E+05	1.03E+05
Ionising radiation	IR	kBq U^235^ eq	3.91E+10	2.45E+10	5.45E+10	3.32E+10	4.80E+10	3.72E+10	5.17E+10	4.20E+10
Human toxicity, cancer	HTOX_c	CTUh	2.21E+04	4.57E+04	3.98E+04	6.00E+04	2.98E+04	6.83E+04	3.48E+04	7.53E+04
Ecotoxicity freshwater	ECOTOX	CTUe	2.89E+12	1.35E+12	3.72E+12	1.76E+12	3.10E+12	1.99E+12	3.50E+12	2.24E+12
Human toxicity, non-cancer	HTOX_nc	CTUh	1.56E+05	1.92E+05	3.06E+05	2.60E+05	2.12E+05	2.88E+05	2.55E+05	3.28E+05
Land use	LU	Pt	2.96E+13	2.07E+13	3.20E+13	2.73E+13	3.24E+13	3.35E+13	3.40E+13	3.74E+13
Water use	WU	m³	6.33E+11	4.51E+11	9.59E+11	6.14E+11	9.01E+11	6.99E+11	9.63E+11	7.72E+11
Resource use, fossils	FRD	MJ	5.37E+13	1.38E+13	6.56E+13	1.68E+13	6.39E+13	1.92E+13	6.32E+13	2.13E+13
Resource use, mineral and metals	MRD	kg Sb eq	2.40E+06	4.27E+06	5.97E+06	5.90E+06	3.76E+06	6.48E+06	4.75E+06	7.40E+06

In the majority of the cases, the environmental impacts caused by imported goods were higher than those caused by exported products as illustrated in Table 2. For four impact categories, i.e. human toxicity non-cancer, freshwater eutrophication, land use, and use of mineral and metals resources, the impacts of exported goods were higher for all the years except 2005. The impacts on human toxicity cancer generated by exported products were higher for all the analysed years.

A contribution analysis was performed to assess the relevance of product groups and substances. Results for 2010 are presented and discussed in sections 3.2.1, 3.2.2.

#### 3.2.1 Contribution analysis of product groups and countries of origin and analysis of temporal trends

**Fig. 2:**
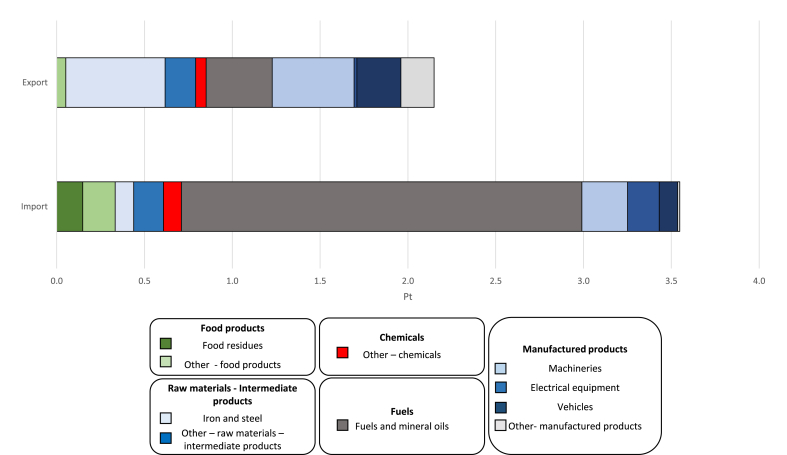
Results expressed as single score (points -Pt) for 2010. Product groups contributing to at least to 3% of the overall single score are reported as self-standing categories, the remaining ones are reported under the label “Other”, which refers to the other product groups listed in Table 1.

Results in Fig. 2 are expressed as a single score (in which the results are aggregated after performing normalisation and weighting) and are grouped according to the product-group categories reported in Table 1. Overall, the impacts of imports were higher than the impacts of exports. “Fuels and mineral oils” was the main drivers of impacts for imports, due to the considerable imported amount, equal to 65% by mass of all the imported products. This was not the case for exports. Although “Fuels and mineral oils” was the most exported product group in terms of mass, it corresponded to 29% by mass of all the exported products and this partly explained its relative lower contribution to the environmental impacts of export. The impact of exports was mainly associated with the product groups “Iron and steel”, “Machineries”, “Fuels and mineral oils”, and "Vehicles".

Products groups falling under the categories “Food products” and “Fuels” presented significant higher impacts for imports than for exports whereas “Manufactured products” and “Raw materials-Intermediate products” were the main drivers of impacts for exported goods. Import of “Raw materials-Intermediate products” by mass was almost double than the exported quantity, but its environmental impact was less than 40% of the one of exported products. This is probably due to the lower complexity of the representative products selected for imports as opposed to exports for the product group “Iron and steel”, as highlighted in Section 3.1.

**Fig. 3:**
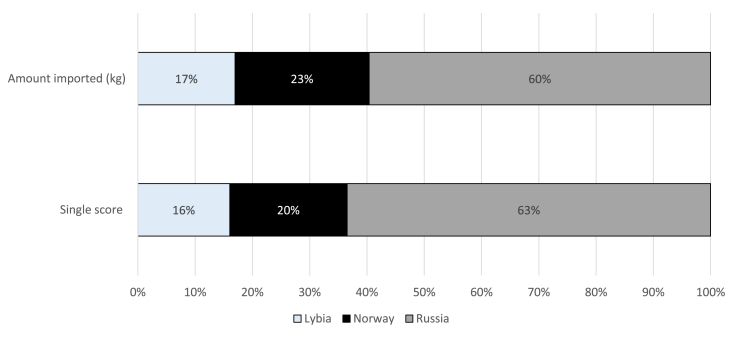
Share of the amount and impacts of imported “Fuels and mineral oils” from the three considered representative countries (year 2010).

The share of impact embodied in imported products coming from different representative countries was investigated. The share of impacts from a country was not always equal to the share of imported mass from that country, highlighting some differences, such as production processes, means of transport, and transport distances. Concerning the product group “Fuels and mineral oils”, for example, imports from Russia represented 60% of the mass of the imported product group, but contributed to between 63% and 97% of the total impacts (depending on the impact category) and accounted for 66% of the single score. Whereas, imports from Norway were 23% of the imported mass but they contributed between 0% and 21% of the total impact (depending on the impact categories) and accounted for 19% of the single score (Fig. 3).

**Fig. 4:**
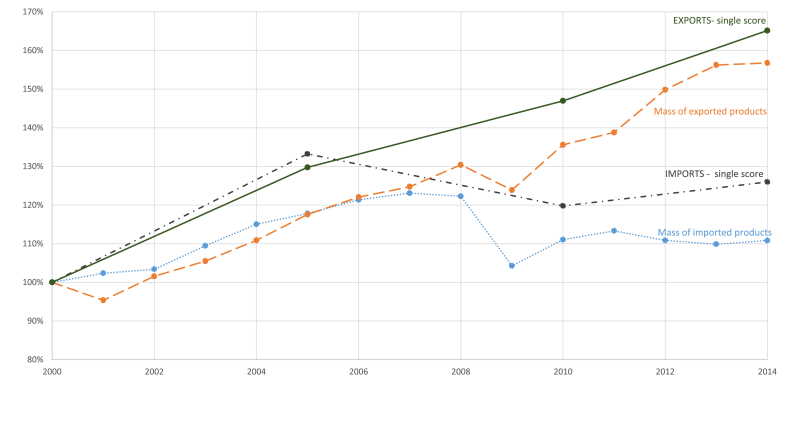
Amounts and environmental impact of imported and exported products. Environmental impacts were calculated for the years 2000, 2005, 2010, 2014, and for the other years are interpolated. Results for 2000 are reported as 100% and the other results are scaled accordingly.

The analysis of temporal trends from 2000 to 2014 showed an increase in the environmental impacts of both imports and exports (Fig. 4). The percentage increase of impacts was higher than the increase in mass for both imports and exports. Knowing that changes in the environmental performances of technologies over time were not considered in the modelling approach adopted, this discrepancy is associated with variations in the type and amount of imported and exported products, and in the case of imported goods, it is due to variations in the countries of origin of different products.

From 2000 to 2005 impacts of imported goods increased by more than 30%. This was mainly due to an increase in imports of the product groups “Fuels and mineral oils”, and “Machineries”, which were hotspots for almost all the impact categories. The observed trend in the imported amount of “Fuels and Mineral oils” influenced as well the decrease of the impact between 2005 and 2010, most likely related to the beginning of the economic crisis, and the increase in impacts from 2010 to 2014.

The evolution of environmental impacts of exported products in the same timeframe was almost linear. The growth of the environmental impacts of exported goods was mainly driven by the increase in terms of mass of those product groups that were the main hotspots for the majority of the impact categories. Between 2000 and 2014, indeed, the exported quantities of “Iron and steel”, “Machineries”, “Fuels and mineral oils”, and “Vehicles” were subject to a double or even bigger increase.

#### 3.2.2 Contribution analysis: impact categories

**Fig. 5:**
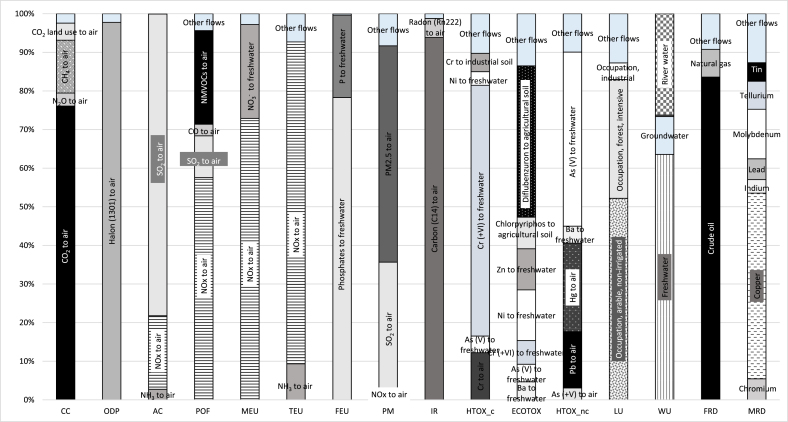
Share of substances for imports for the year 2010. Only substances contributing to more than 3% to impact categories are reported, substances with lower contributions are grouped under the category “other flows”. Acronyms used for impact categories are defined in Table 2. CO_2_: carbon dioxide, N_2_O: dinitrogen monoxide, CH_4_: methane, NH_3_: ammonia, NO_x_: nitrogen oxides, SO_2_: sulphur oxides, NMVOCs: non-methane volatile compounds, NO_3_^−^: nitrates, P: phosphorous, Cr: Chromium, Ni: Nickel, Ba: Barium, Zn: zinc, As: arsenic, Hg: Mercury.

Fig. 5 reports the contribution of substances (emissions and resource use) to each impact category for imported products. The same information for exported goods is reported in the Supplementary materials. The contribution of product groups to each impact category are reported in Table 3 and in Table 4. In both cases a 3% cut-off on the environmental relevance was applied.

**Table 3:** Contribution of imported product groups to environmental impact categories for the year 2010. Only product groups with at least the contribution to one impact category higher than 3% are reported, the share of impact covered by the selected product groups is reported in brackets after the acronym of each impact category. . Acronyms used for impact categories are defined in Table 2.

	Food products					Raw materials - Intermediate products			Chemicals	Fuels	Manufactured products		
	23 Food residues	08 Fruit and nuts	10 Cereals	12 Oilseeds	15 Animal or vegetable fats	26 Ores, slag and ash	47 Pulp of wood or other cellulosic material	72 Iron and steel	28 Inorganic chemicals	27 Fuels and mineral oils	84 Machineries	85 Electrical equipment	87 Vehicles
*Quantity (*100 kg*)*	*3.08E+08*	*1.25E+08*	*9.78E+07*	*1.79E+08*	*1.02E+08*	*1.50E+09*	*1.03E+08*	*3.63E+08*	*1.45E+08*	*1.07E+10*	*9.88E+07*	*8.40E+07*	*6.69E+07*
	%												
CC (92%)	5	0	1	2	2	2	1	8	4	51	4	9	3
ODP (99%)	0	0	0	0	0	0	0	0	0	98	0	0	0
AC (97%)	2	1	1	1	1	3	1	2	0	77	3	3	1
POF (96%)	5	1	1	2	2	6	1	3	0	67	2	3	2
MEU (96%)	12	2	5	9	4	7	2	3	1	45	2	3	1
TEU (94%)	6	1	2	3	4	8	2	4	0	55	3	4	2
FEU (98%)	12	0	1	8	1	0	1	0	0	16	45	8	5
PM (97%)	12	0	0	5	5	6	1	5	0	53	5	3	2
IR (96%)	1	0	0	0	2	1	2	1	3	64	8	6	7
HTOX_c (97%)	3	0	1	1	1	1	1	2	0	36	27	12	12
ECOTOX (98%)	30	8	1	11	1	1	0	0	0	28	11	5	3
HTOX_nc (98%)	2	0	1	1	0	0	1	10	0	28	40	9	6
LU (99%)	24	1	6	24	5	0	26	0	0	12	1	1	0
WU (95%)	1	7	0	0	16	2	5	2	1	40	5	12	4
FRD (97%)	0	0	0	0	0	0	0	1	1	89	1	2	1
MRD (97%)	1	0	0	0	1	0	1	0	0	4	56	25	9

**Table 4:** Contribution of exported product groups to environmental impact categories for the year 2010. Only product groups with at least the contribution to one impact category higher than 3% are reported, the share of impact covered by the selected product groups is reported in brackets after the acronym of each impact category. Acronyms used for impact categories are defined in Table 2.

	Food products	Raw materials - Intermediate products					Chemicals			Fuels		Manufactured products				
	10 Cereals	25 Lime, cements and other materials	39 Plastics	44 Wood and products	47 Pulp of wood or other cellulosic material	72 Iron and steel	28 Inorganic chemicals	29 Organic chemicals	31 Fertilisers	27 Fuels and mineral oils	48 Paper and products	73 Articles of iron or steel	84 Machineries	85 Electrical equipment	87 Vehicles	90 Precision instruments
*Quantity (*100 kg*)*	*2.9E*+*08*	*4.0E*+*08*	*2.0E*+*08*	*2.0E*+*08*	*1.3E*+*08*	*4.8E*+*08*	*1.2E*+*08*	*1.1E*+*08*	*1.2E*+*08*	*1.6E*+*09*	*2.0E*+*08*	*1.2E*+*08*	*1.5E*+*08*	*5.5E*+*07*	*1.4E*+*08*	*5.5E*+*06*
	*%*															
CC (99%)	3	6	8	1	2	17	0	2	1	24	5	6	11	1	10	1
ODP (98%)	4	5	0	0	5	20	1	4	4	0	10	1	18	12	14	1
AC (98%)	5	2	5	0	3	13	7	2	1	18	5	3	20	1	13	1
POF (99%)	3	3	10	1	4	17	1	3	1	20	5	3	13	1	13	1
MEU (94%)	38	2	3	1	3	9	0	1	0	17	4	2	8	1	5	0
TEU (99%)	13	4	5	1	5	15	1	2	1	19	6	3	14	1	9	1
FEU (99%)	3	0	0	0	1	9	0	1	0	3	3	0	68	0	10	0
PM (98%)	3	1	2	6	3	29	2	1	1	7	5	1	24	1	11	2
IR (97%)	1	3	0	1	3	18	0	1	1	8	17	0	19	1	22	1
HTOX_c (100%)	2	0	1	0	1	51	0	0	0	7	1	0	21	0	13	3
ECOTOX (99%)	4	0	1	0	1	38	0	0	0	5	2	0	31	0	12	3
HTOX_nc (100%)	1	1	0	0	1	23	0	0	0	7	2	0	52	0	11	1
LU (99%)	17	0	0	2	35	1	0	0	0	14	27	0	1	0	1	0
WU (98%)	1	1	5	0	9	19	5	2	0	12	16	0	12	1	14	1
FRD (99%)	1	1	9	0	1	8	0	4	1	53	3	2	6	2	7	0
MRD (99%)	1	0	0	0	1	20	1	1	1	1	1	0	59	0	13	2

According to this cut-off, 13 product groups for imports were responsible for over 90% of the impacts, from 92% (climate change) to 99% (land use, ozone depletion). The share of impacts due to exports was more fragmented: 17 product groups were selected, covering about 99% of the impact for all the impact categories.

The contribution of product groups to impact categories was influenced by either the quantity of traded goods, the impact intensities of product groups expressed as impact per unit of mass of product (reported in Supplementary materials), or a combination of the two. In the case of highly imported or exported amounts, relatively high impacts were observed for almost all the impact categories. This was the case, for example, of “Fuels and mineral oils”, which was imported in very high quantity and had a very low impact intensity for all the impact categories, except ozone depletion, but was the main hotspot for almost all the impact categories. On the contrary, hotspots related to particularly high impact intensities were generally limited to few impact categories. For example, the import of “Pulp of wood and other cellulosic material” was 1% of the mass of imported products but was a hotspot for land use due to a land use intensity much higher than the ones of all the other products. It has to be noticed that some product groups, particularly the one selected according to economic value criterion, such as “Pharmaceuticals”, “Aircrafts”, and “Precious materials” had very high impact intensities compared to the other product groups for several impact categories, but their impacts were not relevant in absolute terms due to much lower traded quantities.

“Fuels and mineral oils” exerted, as expected, the highest impact on the use of fossil resources, and presented a higher share of the impacts for imported products (89%) than for exported ones (53%). Crude oil and natural gas represented the two main contributing elementary flows. The importance of crude oil was higher for imported than for exported products, respectively equal to 84% and 62%, due also to the choice of the representative product selected for imports for the products group “Fuels and mineral oils”, which was “crude oil”.

The product group “Fuels and mineral oils” was responsible for 98% of the impact of imported products on ozone depletion, with 97% of the impact coming from Halon 1301 emissions to air. In the case of exported goods, 53% of the impact was due to Halon 1301 emissions to air, mainly generated by “Electrical equipment”, “Machineries”, “Iron and steel”, whereas Halon 1211 emissions, due in large part to “Iron and steel”, “Machineries”, “Vehicles” contributed to 39% of the impact. It should be noted that, in developed countries, Halon 1301 and Halon 1201 have been phased out completely in 2010 according to the Montreal Protocol (United Nations (UN), 1989), with few exceptions. The presence of these emissions in the inventories of traded goods is most likely due to outdated emission factors in secondary datasets, or, in the case of imported products, to different legal prescriptions in force in producing countries.

The impacts on marine and terrestrial eutrophication, and photochemical ozone formation were driven by nitrogen oxides (NO_x_) emissions for both imported and exported goods. Moreover, NO_x_ contributed to a lesser extent also to the impact on acidification. 61% of NO_x_ emissions of imported products were related to “Fossil fuels and mineral oils”, and smaller contributions were from other product groups, e.g. “Food residues” and “Iron and steel”, which were responsible respectively for 4% of total NO_x_ emissions. For exported products, products groups with the highest share of NO_x_ emissions were “Fuels and mineral oils” (18%), “Iron and steel” (17%), “Machineries” (14%), and “Vehicles” (10%).

Imports of product groups related to the food sector, namely “Fruits and Nuts”, “Cereals”, “Oilseeds”, “Animal and vegetable fats”, and “Food residues” were the main responsible for the emissions of ammonia (NH_3_) to air and nitrate (NO_3_^−^) to water, contributing altogether to respectively 70% of the NH_3_ emissions, and 99% of NO_3_^−^ emissions. These emissions were respectively responsible for the impacts on terrestrial eutrophication, acidification, and marine eutrophication. The food sector was less represented in exports, with only one representative product group, i.e. “Cereals”. This explains the considerably lower absolute impacts on acidification (23% of impacts of imported products) and terrestrial eutrophication (33% of the impacts of imported products) of this product group.

For the impact category freshwater eutrophication, the product group “Machineries” had a considerably high impact intensity and was the main hotspot due to emissions of phosphates to water, caused by the disposal of sulphidic tailings, responsible respectively for 42% of the impacts of imported goods and 68% of the impacts of exported products. Exports of "Machineries" were more than 50% higher than imports, explaining the predominance of the impact of exports for freshwater eutrophication. Imported food products contributed overall to 22% of the impact on freshwater eutrophication.

Emissions of sulphur dioxide (SO_2_) represented the main contributor to acidification and influenced to a lesser extent the impact categories photochemical ozone formation and particulate matter. “Fuels and mineral oils” generated 84% of SO_2_ emissions of imports, and 17% of SO_2_ emissions of exports. Other product groups causing relevant SO_2_ emissions in the case of exports were “Vehicles” (16% of the emissions), “Inorganic chemicals” (10% of the emissions), and “Plastics” (5% of the emissions).

The impact category particulate matter was driven by emissions of fine particulate matter (PM_2.5_) both in the case of imported and exported goods. These emissions were mainly generated by, respectively, “Fuels and mineral oils”, responsible for 35% of PM_2.5_ emissions of imported products, and “Iron and steel”, generating 34% of emissions of exported products.

The main contributors to the impact on climate change of imported goods were “Fuels and mineral oils” (51%), “Electrical equipment” (9%), and “Iron and Steel” (8%). With a share of 76% of the total impact, carbon dioxide (CO_2_) was by far the most important substance emitted, followed by methane (CH_4_) (14%), CO_2_ from land transformation (4%), and nitrous oxide (N_2_O) (3%). In the case of exports, “Fuels and mineral oils” was responsible for 24% of the impact on climate change, followed by “Iron and Steel” (17% of the impact), “Machineries” (11%), and “Vehicles” (10%). The main contributing substances were CO_2_ (83%) and CH_4_ (12%). The contributions of N_2_O and CO_2_ due to land transformation - mainly associated with food products, not prominent within exported goods - were under the 3% cut-off.

Toxicity-related impact categories were mainly influenced by emissions of heavy metals in the environment for both imports and exports. At a first glance, emissions of zinc to various environmental compartments were driving the impacts on human toxicity non-cancer, and the underpinning motivation was thoroughly investigated. Characterisation factors for zinc emissions for the above-mentioned impact category were calculated considering emission, fate, exposure, effect, and damage caused by the emitted substance. When looking at the intake values for metals emitted to agricultural soil, the highest value for zinc is found for the ingestion via exposed produce (i.e. above-ground leaf crops). However, there are several documents highlighting the problem of zinc deficiency in soils (Alloway, 2008), and related human health problems due to zinc deficiency. In order to take this situation into account, the developers of the impact assessment method IMPACT World+, who based the assessment of the impact on toxicity on the USEtox model (which also underpins the EF 1.8 method), modified the original characterisation factors assuming that only 2% of the world population is really at risk of toxicity effects related to zinc intake (Olivier Jolliet, personal communication). The same approach was followed in this study, where the characterisation factors for the impact category human toxicity, non-cancer for zinc emissions to all the environmental compartments were reduced by 98%. The absolute impacts on human toxicity was higher for exported than for imported products, and, concerning the first ones, exported “Iron and Steel” and “Machineries” were the two main hotspots for human toxicity respectively cancer and non-cancer. This was essentially due to a combination of high impact intensities of the hotspot product groups compared to the others and higher exported amounts. Almost half (47%) of the impact on ecotoxicity of imported goods was caused by the emissions of two pesticides to the soil, namely Diflubenzuron and Chlorpyrifos, mainly due to the product groups “Food residues”, and “Animal and vegetable fats”. Conversely, the impact of exported products on ecotoxicity, was driven by emissions of heavy metals, mainly nickel and Chromium VI to freshwater from “Iron and steel”. This, once again, highlights that imported food products presented significantly higher impacts than exported ones.

The product group “Pulp of wood and other cellulosic material” was a hotspot for land use for both imported and exported products, due to a high impact intensity. Other relevant contributions to land use for imported goods were from food products, which altogether contributed to about 60% of the overall impact. In the case of exported goods, “Paper and products” contributed to 27% of the impact, followed by “Cereals” (17%) and “Fuels and mineral oils” (14%).

The impact on mineral and metal resources use was driven by copper both in the case of imported and exported products, followed by molybdenum. The use of copper and molybdenum was to a greater extent associated with the product group “Machineries”, responsible of more than 55% of the impacts for both imported and for exported products. However, the results have been calculated with the Abiotic Depletion Potential characterisation model (using the ultimate reserves set of characterisation factors) (Guinée et al., 2002; van Oers et al., 2002) that characterises a limited set of resources.

The extent to which product groups were contributing to water use was different between imported and exported products. “Fuels and mineral oils” were the main hotspot for imported goods (40% of the impact), followed by “Animals and vegetable fats” (16%), and “Electrical equipment” (12%). In the case of exported goods, main concerns were “Iron and steel” (19%), “Paper and products” (16%), “Vehicles” (14%), “Machineries” (12%), and “Fuels and mineral oils” (12%).

Ionising radiation was mainly influenced by “Fuels and mineral oils” in the case of imported products, which caused 64% of the impact. Within exported products, the main contributors were the product groups “Vehicles”, “Machineries”, and “Iron and steel”, with a share of respectively 22%, 19% and 18% of the impact.

### 3.3 Contribution of traded products to EU environmental impacts

In order to explore the extent to which traded goods are contributing to the overall environmental impacts of consumption in the EU, the impacts of imported and exported products were compared with the ones generated domestically in the EU and the impacts of imported goods were compared with the ones of EU apparent consumption (Table 5).

**Table 5:**
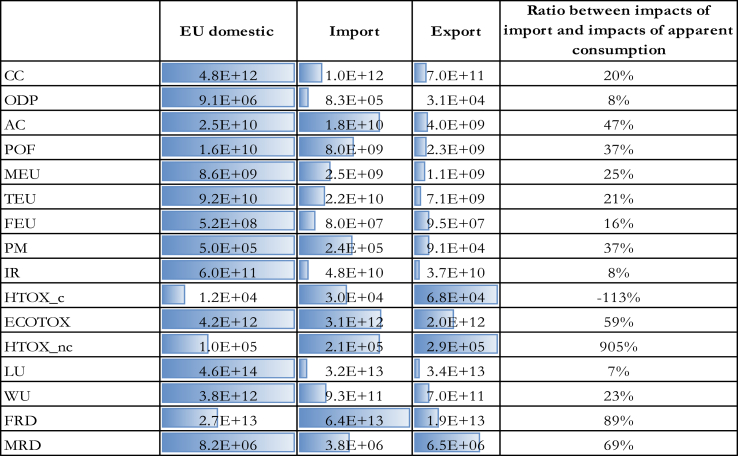
Comparison of the impacts of import, export, and domestic production for the year 2010. Acronyms used for impact categories are defined in Table 2.

The way in which apparent consumption is calculated could lead to a sort of paradoxical “export effect”, according to which the higher is the impact of exported products the lower is the impact of EU apparent consumption. This was particularly relevant for some impact categories such as human toxicity non-cancer, where the significant impacts of exported products play a considerable role in contributing to reduce the burdens of EU apparent consumption, while increasing the overall environmental burdens exerted on the global environment. An anomalous situation was observed for the impact category human toxicity cancer that had a negative value for apparent consumption. This situation cannot happen in reality unless the exported products were produced in previous years and were part of the stock that is not considered in this study. Having verified that the apparent consumption for human toxicity cancer was negative for all the analysed years, it was considered very likely that the negative results were due to inconsistencies in the accounting approach highlighted in the following paragraph, rather than to the export of products pertaining to the EU stock. Therefore, the results on human toxicity cancer for apparent consumption were excluded from further considerations on the results.

These critical results related to apparent consumption should be interpreted taking into consideration the combined effect of the limitations of the accounting approach adopted. Indeed, it has to be considered that the differences in the approaches adopted to calculate the impacts of traded goods and domestic activities may affect the type of emission considered and their disaggregation. A thorough review of the elementary flows mapping^1^ was performed to assure consistency in the characterisation of elementary flows. However, the lack of detailed information on some of the elementary flows in one or in the other approach may limit the meaningfulness of the comparison, particularly for those impact categories in which several flows are contributing significantly to the overall impacts, such as toxicity-related ones, resource use, and land use. In addition, the estimation of the impacts of the apparent consumption might be affected by: (i) uncertainties in data used to assess the environmental burdens of both domestic activities, and imported and exported products, (ii) the criteria adopted for the selection of representative products, namely mass and economic value, (iii) the upscaling procedure, and, as highlighted before, (iv) the exclusion of the stock from the accounting may have led to biased estimations of the impacts of apparent consumption. Despite these possible methodological flaws, the analysis of the apparent consumption was deemed relevant to make some considerations on the environmental impacts of traded goods.

The extent to which imports were contributing to EU apparent consumption was analysed considering the ratio between the impact of imported products and apparent consumption (Table 5). Low ratios between impacts of imported products and apparent consumption mean that the impact was generated mainly domestically and was associated just to a small extent with the exported products. On the contrary, high ratios identify situations in which the impact of imported and/or exported products was more relevant. Ratios higher than 100% are associated with impact categories for which the impact of imports was higher than the impact of apparent consumption, and negative ratios are due to situations in which the impact of exports was higher than the one of the sum of domestic plus imports (negative apparent consumption).

Human toxicity non-cancer had a peculiar situation in which both the impacts of imported and exported goods were high compared to the ones of domestic activities. Two possible reasons for this are that: i) human toxicity non-cancer was driven in both cases by “Machineries” product group, for which only few transformations are performed in the EU, and ii) the estimation of the impact on toxicity in the LCA field is characterised by a high level of uncertainty (Zampori et al., 2016). Except for human toxicity cancer and non-cancer, the ratio between the impacts of imported products and apparent consumption varied between 7% and 89%.

Most of the impact on fossil and mineral resource use was generated outside EU boundaries, reflecting the relatively low extraction activity of minerals and metals, and fossil fuels taking place in the EU (Eurostat, 2018). The impact of imported products was relevant also for the impact categories particulate matter, photochemical ozone formation, acidification, and freshwater ecotoxicity. On the contrary, the impact of imported products on ionising radiation was very limited compared to the impacts of domestic activity, highlighting that a high share of nuclear energy at the global level is produced within EU boundaries (Crenna et al., 2019).

Results on land use are in contrast with results from other studies found in the literature (Meier et al., 2014 for Germany; Wiedmann et al., 2007). This may be due to inconsistencies between the inventories of domestic activities and traded goods, as explained before.

### 3.4 Contribution of traded products to global environmental impacts

**Fig. 6:**
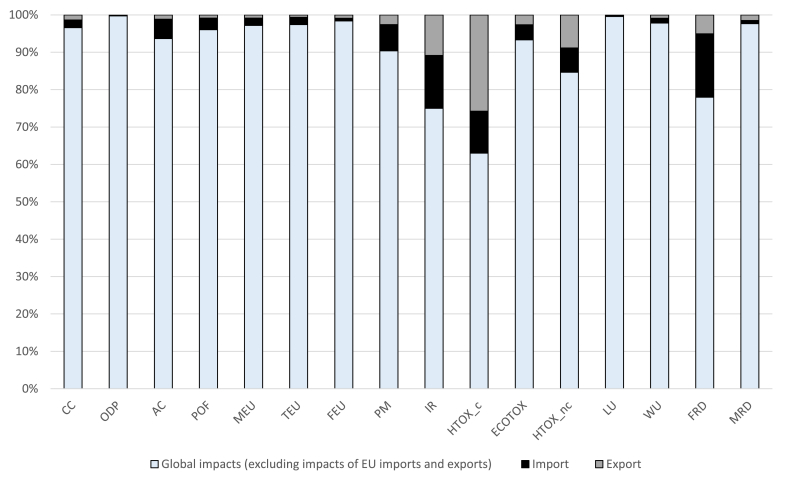
Share of the global environmental impacts due to EU imports and exports. Acronyms used for impact categories are defined in Table 2.

In order to investigate the extent to which EU trade contributed to global environmental burdens, the impacts of imported and exported products were compared with the global normalisation factors updated from Sala et al. (2017) (Fig. 6), who estimated the overall impacts generated at global level, considering the 16 EF impact categories.

The impacts of the sum of EU imported and exported products were contributing to more than 5% to global impacts for seven of the 16 impact categories analysed, i.e. acidification, particulate matter, ionising radiation, human toxicity cancer and non-cancer, freshwater ecotoxicity, use of fossil resources. Peaks of more than 20% were observed for human toxicity, cancer (37%), ionising radiation (25%), and use of fossil resources (22%). Exported products represented a higher contribution to the human toxicity impact categories (cancer and non-cancer) than imported ones. Emissions of heavy metals were the main contributors to these impact categories and the predominance of exported on imported goods can be explained considering that: (i) exports of “Manufactured products” (the main responsible for such emissions) were larger than imports, and (ii) the representative products in the category “Manufactured products” for exports presented higher impact intensities than those selected for imports. Impacts of imported products were predominant for the impact category use of fossil resource, representing 17% of the global share. This is explained by the EU reliance on imports for fossil fuels supply. The relevance of the ionising radiation for imports is mainly associated with the production of "Fuels and mineral oils". For exports, instead, various energy-intensive product groups significantly contributed to the impact on ionising radiation, such as "Vehicles", "Machineries", and "Iron and steel". This is due to the considerable share of the global electricity production from nuclear taking place within the EU boundaries, as discussed above.

### 3.5 Limitations and outlook of the study

The present study, based on process-based LCA, provides a more detailed picture on the environmental impacts of trade compared to studies based on EEIOTs. Anyway, it is affected by a number of limitations, mainly related to the method adopted to choose representative products and to upscale the results to overall imported and exported products, which are discussed in the following paragraphs. Building on the findings of this study and the experience of Lavers et al. (2017), who proposed a method to select representative products for quantifying environmental impacts of consumption in urban areas, possible ways to overcome these limitations in future updates of the study are here presented.

Firstly, assuming that a single product is representative of an entire product group added a high level of approximation to the study, particularly for product groups that encompass a broad set of products with different characteristics. Second, the selection of the product groups was originally based on mass and economic value reported in statistics for 2010. The selection of representative products within each product group, instead, was done exclusively on a mass basis, for the same year. As highlighted by Lavers et al. (2017) this approach may underpin two main weaknesses. The first is that mass and economic value may not be the best predictors of environmental impact. For example, clothes are largely imported by the EU and are deemed to contribute significantly to specific environmental impacts of consumption (Pedersen and Andersen, 2015; House of Commons Environmental Audit Commitee, 2019). However they were not included in the product categories when applying the the mass and economic criteria. The second is that the contribution of representative products to the environmental burdens of EU consumption may change over time.

In order to overcome these limitations, the environmental relevance of products should be considered as selection criteria for products groups and representative products. A recent study by Beylot et al. (2019), comparing bottom-up (process based LCA) and top-down (EEIOT) approaches to assess impact of traded goods, revealed that complementing the selection of product groups in mass and economic values with the product groups driving the results of e.g. EEIOT may help completing the assessment. For example, adding representative products among products groups such as meat and textile may increase the coverage of products and improve the assessment of the impacts. This could lead to the development of an hybrid-LCA framework with the advantage of combining the broad scope of EEIOTs with the detailed results achievable through process based LCA. Furthermore, to ensure a higher representativeness of the selected product groups, the number of representative products per each product group should be increased in such a way that they cover at least 50% of the mass of the product groups (Lavers et al., 2017) and the selection of representative products should be year-specific. This is important when a broad timespan is analysed, i.e. more than 20 years, and the type of imported and exported products may change over time.

Other limitations regarding LCI data availability were the missing accounting of technological changes and the limited availability of country-specific and regionalised LCIs. Indeed, in a timeframe of almost 15 years, technological changes may happen due to, e.g. efficiency improvements or new legal requirements, and significant differences in local environmental regulation or technological development from country to country may determine a variation in the emission intensities associated with a certain production. Moreover, some environmental impacts, such as land use and water use, have a local nature and the production of one good can cause different environmental impacts, depending on the vulnerability of the area where the good is produced. Hence, having regionalised elementary flows may be particularly relevant for impact categories reflecting local to regional impacts. Although it might be arduous to capture all these elements in secondary LCIs, they should be considered as far as possible in future updates of the study.

Lastly, the analysis of other studies found in literature highlighted some relevant environmental concerns related to trade, e.g. biodiversity loss in developing countries, for which a robust and systematised assessment framework is not yet available for LCA. Therefore, a comprehensive assessment of the impacts of trade should encompass elements, which are currently only partially captured by the LCA framework.

## 4 Conclusions

The analysis of the environmental performance of the products traded by the EU highlighted that the impacts of imported goods are higher than the ones of exported products for the majority of the analysed impact categories, emphasizing that a share of the impacts associated with EU consumption is taking place outside EU boundaries. Specifically, EU mainly imports “Fuels”, and “Raw materials-Intermediate products”, and exports “Manufactured products”. Few product groups were found to be relevant contributors to several impact categories: “Fuels and mineral oils” for imports, and “Iron and steel”, “Machineries”, and “Vehicles” for exports. The environmental impacts of food imports was higher than the one of food exports. The impact of imports and exports of food was mainly related to specific emissions, such as ammonia, nitrous oxide, and carbon dioxide from land transformation to air, nitrates to water, and pesticide to soil, where this sector was predominant.

The comparison between the environmental performance of trade and domestic activities showed that imports are a key contributor for some impact categories, such as fossil resource depletion, human toxicity non-cancer, acidification, and freshwater ecotoxicity.

Imported quantities and impact intensities were the drivers of environmental impacts. Effective strategies to reduce the absolute environmental impacts of consumption associated with imported products, therefore, may in principle encompass a decrease in the use of highly imported products (e.g. “Fuels and mineral oils”), as well as an optimisation of the production process of imported products with high impact intensities (e.g. “Pulp of wood and other cellulosic material” for land use) on which the EU can only exert an indirect control.

The comparison between the environmental impacts of traded goods and the global impacts highlighted that EU trade is particularly contributing to human toxicity cancer (particularly relevant for exports from the EU), ionising radiation (relevant for both imports and exports), and use of fossil resources (particularly relevant for imports to the EU).

Overall, traded products represent only a fraction of the goods consumed in the EU and solutions for environmentally friendly consumption patterns should encompass an improvement in production efficiency as well as a substantial shift towards more sustainable consumption patterns (Bjørn et al., 2018; Sala et al., 2019).

From the methodological point of view, this study presents some limitations, which may be overcome in future updates of the assessment. The inclusion of environmental relevance of products as selection criteria and an increase in the number of representatives products analysed within each product groups were identified as priorities to improve the reliability of the assessment. In addition, some of the impacts traditionally imputed to EU imports, e.g. loss of biodiversity, were here addressed by means of the midpoint impact categories determining biodiversity loss (e.g. climate change, land use etc). However, to reach a comprehensive overview of the environmental burdens of trade, there is the need to broaden the scope of the analysis beyond traditional LCA impact categories.

## Declaration of competing interest

The authors declare that they have no known competing financial interests or personal relationships that could have appeared to influence the work reported in this paper.

## Footnotes

1 The term “mapping” here refers to the exercise of defining a correspondence between the names of elementary flows in original life cycle inventories used for trade components and for EU domestic activities and the names of the elementary flows reported in the reference characterisation method, i.e. EF 1.8. This procedure is essential to ensure a comprehensive characterisation of elementary flows.
