# Supplementary material for: Out of sight out of mind? A life cycle-based environmental assessment of goods traded by the European Union
Source: J Clean Prod. 2020 Feb 10;246:118954. doi: 10.1016/j.jclepro.2019.118954 (PMC6961971; doi:10.1016/j.jclepro.2019.118954)
Supplement: Multimedia component 1 [file mmc1.docx]

**Out of sight out of mind? A life cycle based environmental assessment of goods traded by the European Union**

Sara Corrado^a^, Tomas Rydberg^b^, Felipe Oliveira^b^, Alessandro Cerutti^a^, Serenella Sala^a^

^a^ European Commission-Joint Research Centre, Via Enrico Fermi 2749, I-21027 Ispra (VA), Italy

^b^ IVL Swedish Environmental Research Institute, 100 31, Stockholm, Sweden

*corresponding author: serenella.sala@ec.europa.eu

**Contents**

1. Product groups and representative products
2. Ratio between imports and exports, years 2000, 2005, 2010, and 2014
3. Coverage of representative products and representative importing countries
4. Impact intensities
5. Contribution of substances
6. Normalization and weighting factors
7. **Product groups and representative products**

Table 1 reports a list of the extensive names of product groups and representative products considered for the analysis of the environmental impacts of trade.

Table 1: Extensive name of products groups and representative products as reported in Eurostat database (EU trade since 1988 by HS2, 4, 6 and CN8 (DS-645593))

| **Product group** | **Representative product - IMPORT** | **Representative product - EXPORT** |
| --- | --- | --- |
| 08 - Edible Fruit And Nuts; Peel Of Citrus Fruits Or Melons | 08030019 - bananas, fresh (excl. Plantains) |  |
| 10 - Cereals | 10059000 - maize (excl. Seed for sowing) | 10019099 - Spelt, common wheat and meslin (excl. Seed) |
| 12 - Oil Seeds And Oleaginous Fruits; Miscellaneous Grains, Seeds And Fruit; Industrial Or Medicinal Plants; Straw And Fodder | 12010090 - soya beans (excl. For sowing) |  |
| 15 - Animal Or Vegetable Fats And Oils And Their Cleavage Products; Prepared Edible Fats; Animal Or Vegetable Waxes | 15111090 - crude palm oil (excl. For technical or industrial uses) |  |
| 23 - Residues And Waste From The Food Industries; Prepared Animal Fodder | 23040000 - oilcake and other solid residues, whether or not ground or in the form of pellets, resulting from the extraction of soya-bean oil |  |
| 25 - Salt; Sulphur; Earths And Stone; Plastering Materials, Lime And Cement | 25171080 - broken or crushed stone, for concrete aggregates, for road metalling or for railway or other ballast, whether or not heat-treated (excl. Pebbles, gravel, flint and shingle, broken or crushed dolomite and limestone flux) | 25232900 - Portland cement (excl. White, whether or not artificially coloured) |
| 26 - Ores, Slag And Ash | 26011100 - non-agglomerated iron ores and concentrates (excl. Roasted iron pyrites) | 26011200 - Agglomerated iron ores and concentrates (excl. Roasted iron pyrites) |
| 27 - Mineral Fuels, Mineral Oils And Products Of Their Distillation; Bituminous Substances; Mineral Waxes | 27090090 - petroleum oils and oils obtained from bituminous minerals, crude (excl. Natural gas condensates) | 27101141 - Motor spirit, with a lead content <= 0,013 g/l, with an research octane number "RON" of < 95 |
| 28 - Inorganic Chemicals; Organic Or Inorganic Compounds Of Precious Metals, Of Rare-Earth Metals, Of Radioactive Elements Or Of Isotopes | 28141000 - anhydrous ammonia | 28070010 - Sulphuric acid |
| 29 - Organic Chemicals | 29051100 - methanol "methyl alcohol" | 29091990 - Acyclic ethers and their halogenated, sulphonated, nitrated or nitrosated derivatives (excl. Diethyl ether and tert-butyl ethyl ether [ethyl-tertio-butyl-ether, ETBE]) |
| 30 - Pharmaceutical products |  | 30049000 - Medicaments consisting of mixed or unmixed products for therapeutic or prophylactic purposes, put up in measured doses "incl. Those in the form of transdermal administration" or in forms or packings for retail sale (excl. Medicaments containing antibiotics, medicaments containing hormones or steroids used as hormones, but not containing antibiotics, medicaments containing alkaloids or derivatives thereof but not containing hormones or antibiotics and medicaments containing provitamins, vitamins or derivatives thereof used as vitamins) |
| 31-Fertilisers | 31021010 - urea, whether or not in aqueous solution, containing > 45% nitrogen in relation to the weight of the dry product (excl. That in pellet or similar forms, or in packages with a gross weight of <= 10 kg) | 31022100 - Ammonium sulphate |
| 39 - Plastics And Articles Thereof | 39012090 - Polyethylene with a specific gravity of >= 0,94, in primary forms (excl. Polyethylene in blocks of irregular shape, lumps, powders, granules, flakes and similar bulk forms, of a specific gravity of >= 0,958 at 23°c, containing <= 50 mg/kg of aluminium, <= 2 mg/kg of calcium, of chromium, of iron, of nickel and of titanium each and <= 8 mg/kg of vanadium, for the manufacture of chlorosulphonated polyethylene) | 39012090 - Polyethylene with a specific gravity of >= 0,94, in primary forms (excl. Polyethylene in blocks of irregular shape, lumps, powders, granules, flakes and similar bulk forms, of a specific gravity of >= 0,958 at 23°C, containing <= 50 mg/kg of aluminium, <= 2 mg/kg of calcium, of chromium, of iron, of nickel and of titanium each and <= 8 mg/kg of vanadium, for the manufacture of chlorosulphonated polyethylene) |
| 44 - Wood And Articles Of Wood; Wood Charcoal | 44039959 - birch, in the rough, whether or not stripped of bark or sapwood, or roughly squared (excl. Sawlogs; rough-cut wood for walking sticks, umbrellas, tool shafts and the like; wood cut into boards or beams, etc.; wood treated with paint, stains, creosote or other preservatives) | 44071091 - Spruce "Picea abies Karst." or silver fir "Abies alba Mill.", sawn or chipped lengthwise, sliced or peeled, of a thickness of > 6 mm (excl. Planed, sanded or end-jointed; boards for the manufacture of pencils; wood with a length of <= 125 mm and a thickness of < 12,5 mm) |
| 47 - Pulp Of Wood Or Of Other Fibrous Cellulosic Material; Recovered (Waste And Scrap) Paper Or Paperboard | 47032900 - semi-bleached or bleached non-coniferous chemical wood pulp, soda or sulphate (excl. Dissolving grades) | 47032100 - Semi-bleached or bleached coniferous chemical wood pulp, soda or sulphate (excl. Dissolving grades) |
| 48 - Paper And Paperboard; Articles Of Paper Pulp, Of Paper Or Of Paperboard |  | 48101990 - Paper and paperboard used for writing, printing or other graphic purposes, not containing fibres obtained by a mechanical or chemi-mechanical process or of which <= 10% by weight of the total fibre content consists of such fibres, coated on one or both sides with kaolin or other inorganic substances, in square or rectangular sheets with one side > 435 mm or with one side <= 435 mm and the other side > 297 mm in the unfolded state (excl. Those used as a base for photosensitive, heat-sensitive or electrosensitive paper or paperboard weighing <= 150 g/m²) |
| 71 - Natural or cultured pearls, precious or semi-precious stones, precious metals, metals clad with precious metal, and articles thereof; imitation jewellery; coin | 71179000 - imitation jewellery (excl. Jewellery, of base metal, whether or not clad with silver, gold or platinum) | 71181090 - Coin (excl. Coin being legal tender, gold and silver coin, medals, jewellery of coins, collectors'' coins, waste and scrap) |
| 72-Iron and steel | 72071210 - semi-finished products of iron or non-alloy steel, containing by weight < 0,25 of carbon, of rectangular "other than square" cross-section, the width measuring >= twice the thickness, rolled or obtained by continuous casting | 72142000 - Bars and rods, of iron or non-alloy steel, with indentations, ribs, groves or other deformations produced during the rolling process |
| 73 - Articles Of Iron Or Steel |  | 73051100 - Line pipe of a kind used for oil or gas pipelines, having circular cross-sections and an external diameter of > 406,4 mm, of iron or steel, longitudinally submerged arc welded |
| 84 - Nuclear Reactors, Boilers, Machinery And Mechanical Appliances; Parts Thereof | 84099900 - parts suitable for use solely or principally with compression-ignition internal combustion piston engine "diesel or semi-diesel engine", n.e.s. | 84314980 - Parts of machinery of heading 8426, 8429 and 8430, n.e.s. |
| 85 - Electrical machinery and equipment and parts thereof; sound recorders and reproducers, television image and sound recorders and reproducers, and parts and accessories of such articles | 85414090 - photosensitive semiconductor devices, incl. Photovoltaic cells | 85451910 - Electrodes of graphite or other carbon, for electrical purposes (excl. Those for electrolysis installations or furnaces) |
| 87 - Vehicles Other Than Railway Or Tramway Rolling-Stock, And Parts And Accessories Thereof | 87032210 - motor cars and other motor vehicles principally designed for the transport of persons, including station wagons and racing cars, with spark-ignition internal combustion reciprocating piston engine of a cylinder capacity > 1 000 cc but =< 1.500 cc, new (excl. Vehicles for the transport of persons on snow and other specially designed vehicles of subheading no 8703.10) | 87032319 - Motors caravans with spark-ignition internal combustion reciprocating piston engine, of a cylinder capacity > 1.500 cm³ but <= 3.000 cm³, new |
| 88 - Aircraft, spacecraft, and parts thereof |  | 88024000 - Aeroplanes and other powered aircraft of an of an unladen weight > 15.000 kg (excl. Helicopters and dirigibles) |
| 90 - Optical, photographic, cinematographic, measuring, checking, precision, medical or surgical instruments and apparatus; parts and accessories thereof | 90191090 - mechano-therapy appliances; massage apparatus; psychological aptitude-testing apparatus (excl. Electrical vibratory-massage apparatus) | 90189085 - Instruments and appliances used in medical, surgical or veterinary sciences, n.e.s. |

1. **Ratio between imports and exports, years 2000, 2005, 2010, and 2014**

Table 2 reports the ration between the impact of imports and the impacts of exports for the years 2000, 2005, 2010, and 2014. Results higher than 100% are typical of situations in which the impact of imports is higher than the one of exports. On the contrary, ratios lower than 100% indicates cases in which the impacts of exports are higher in respect to the ones of imports.

Table 2: Ratio between imports and exports for the years 2000, 2005, 2010 and 2014.

| **Impact category** | **Acronym** | **Unit** | **2000** | **2005** | **2010** | **2014** |
| --- | --- | --- | --- | --- | --- | --- |
| Climate change | CC | kg CO_2_ eq | 174% | 178% | 147% | 138% |
| Ozone depletion | ODP | kg CFC-11 eq | 3337% | 3068% | 2706% | 2222% |
| Acidification | AC | molc H^+^ eq | 550% | 534% | 444% | 412% |
| Photochemical ozone formation | POF | kg NMVOC eq | 448% | 426% | 352% | 321% |
| Eutrophication, marine | MEU | kg N eq | 270% | 321% | 236% | 208% |
| Eutrophication, terrestrial | TEU | molc of N eq | 381% | 384% | 307% | 278% |
| Eutrophication, freshwater | FEU | kg P eq | 97% | 137% | 85% | 88% |
| Particulate matter | PM | disease incidences | 351% | 332% | 267% | 254% |
| Ionising radiation | IR | kBq U^235^ eq | 160% | 164% | 129% | 123% |
| Human toxicity, cancer | HTOX_c | CTUh | 48% | 66% | 44% | 46% |
| Ecotoxicity freshwater | ECOTOX | CTUe | 213% | 212% | 155% | 157% |
| Human toxicity, non-cancer | HTOX_nc | CTUh | 81% | 117% | 74% | 78% |
| Land use | LU | Pt | 143% | 117% | 97% | 91% |
| Water use | WU | m³ | 140% | 156% | 129% | 125% |
| Resource use, fossils | FRD | MJ | 389% | 391% | 333% | 297% |
| Resource use, mineral and metals | MRD | kg Sb eq | 56% | 101% | 58% | 64% |

1. **Coverage of representative products and representative importing countries**

Table 3 and Table 4 provide an overview of the extent to which each representative product is representative of the related product groups, and, in the case of imported products, to which extent representative countries cover the imports from all the importing countries. The representativeness is expressed in terms of mass.

Table 3: Representativeness of imported representative products within the product groups and of representative countries. Referred to year 2010 (% referred to the mass of products)

| **Product group** | **Share of the representative product on the product group** | **Share from representative countries on the representative product** | **Representative countries and share of products** | | |
| --- | --- | --- | --- | --- | --- |
| 08 | 36% | 70% | Colombia | Costa Rica | Ecuador |
|  |  |  | 26% | 17% | 28% |
| 10 | 38% | 73% | Brazil | Serbia | Ukraine |
|  |  |  | 43% | 16% | 15% |
| 12 | 75% | 84% | USA | Brazil | Paraguay |
|  |  |  | 22% | 44% | 18% |
| 15 | 28% | 94% | Indonesia | Malaysia | Papua Nuova Guinea |
|  |  |  | 44% | 34% | 15% |
| 23 | 71% | 98% | USA | Brazil | Argentina |
|  |  |  | 5% | 42% | 51% |
| 25 | 14% | 105% | Norway | Ukraine | Croatia |
|  |  |  | 86% | 10% | 10% |
| 26 | 60% | 76% | Ukraine | Canada | Brazil |
|  |  |  | 12% | 8% | 56% |
| 27 | 48% | 59% | Norway | Russia | Libyan Arab Jamahiriya |
|  |  |  | 14% | 35% | 10% |
| 28 | 17% | 90% | Russia | Algeria | Egypt |
|  |  |  | 57% | 26% | 7% |
| 29 | 31% | 40% | Russia | Trinidad Tobago | Saudi Arabia |
|  |  |  | 17% | 13% | 11% |
| 31 | 23% | 87% | Russia | Libyan Arab Jamahiriya | Egypt |
|  |  |  | 28% | 9% | 51% |
| 39 | 9% | 67% | Iran | Saudi Arabia | Qatar |
|  |  |  | 19% | 40% | 8% |
| 44 | 11% | 99% | Ukraine | Belarus | Russia |
|  |  |  | 3% | 20% | 76% |
| 47 | 54% | 86% | Brazil | Chile | Uruguay |
|  |  |  | 62% | 12% | 11% |
| 71 | 29% | 92% | India | China | Hong Kong |
|  |  |  | 6% | 82% | 4% |
| 72 | 17% | 97% | Ukraine | Russia | Brazil |
|  |  |  | 43% | 51% | 3% |
| 84 | 3% | 49% | USA | Brazil | China |
|  |  |  | 14% | 22% | 14% |
| 85 | 16% | 82% | USA | Malaysia | China |
|  |  |  | 5% | 10% | 67% |
| 87 | 9% | 62% | Turkey | Japan | India |
|  |  |  | 20% | 18% | 24% |
| 90 | 12% | 96% | USA | Canada | China |
|  |  |  | 8% | 3% | 85% |

Table 4. Representativeness of exported representative products within the product groups. Referred to year 2010 (% referred to the mass of products)

| **Product group** | **Share of the representative product on the product group** |
| --- | --- |
| 10 | 75% |
| 25 | 19% |
| 26 | 65% |
| 27 | 14% |
| 28 | 20% |
| 29 | 4% |
| 30 | 66% |
| 31 | 14% |
| 39 | 6% |
| 44 | 16% |
| 47 | 12% |
| 48 | 9% |
| 71 | 34% |
| 72 | 6% |
| 73 | 14% |
| 84 | 3% |
| 85 | 9% |
| 87 | 17% |
| 88 | 50% |
| 90 | 8% |

1. **Impact intensities**

Table 3 and Table 4 report the impact intensities for each impact category, expressed as impact per 100 kg of products. The impact intensities were calculated by dividing the results of 2010 by the amount of imported and exported products as reported in Eurostat. Differences between imports and exports are explained by the choice of different representative products in most of the case, and by different transport distances (transport of the final products to the extra-EU importing countries is not considered in the export)

Table 5: Impact intensities, expressed as impact per 100 kg of product, calculated for imports in 2010

|  | 71-Precious materials | 84-Machineries | | | 85-Electrical equipment | | 87-Vehicles | | 90-Precision instruments | | 08-Fruit and nuts | | 10-Cereals | | 12-Oilseeds | | 15-Animal or vegetable fats | | 23-Food residues |
| --- | --- | --- | --- | --- | --- | --- | --- | --- | --- | --- | --- | --- | --- | --- | --- | --- | --- | --- | --- |
| CC | 8.9E+02 | 4.1E+02 | | | 9.7E+02 | | 4.3E+02 | | 4.2E+02 | | 3.3E+01 | | 6.8E+01 | | 1.2E+02 | | 2.0E+02 | | 1.6E+02 |
| ODP | 1.4E-06 | 2.9E-05 | | | 3.3E-05 | | 2.5E-05 | | 2.5E-05 | | 1.3E-06 | | 4.1E-06 | | 1.8E-06 | | 5.2E-06 | | 3.1E-06 |
| AC | 4.8E+00 | 4.3E+00 | | | 6.9E+00 | | 3.6E+00 | | 3.8E+00 | | 6.7E-01 | | 1.1E+00 | | 1.1E+00 | | 2.0E+00 | | 1.2E+00 |
| POF | 2.8E+00 | 1.6E+00 | | | 3.0E+00 | | 2.2E+00 | | 2.2E+00 | | 4.0E-01 | | 4.1E-01 | | 8.9E-01 | | 1.8E+00 | | 1.3E+00 |
| MEU | 9.7E-01 | 4.8E-01 | | | 9.0E-01 | | 4.7E-01 | | 4.9E-01 | | 3.5E-01 | | 1.2E+00 | | 1.1E+00 | | 8.7E-01 | | 9.3E-01 |
| TEU | 1.1E+01 | 5.5E+00 | | | 1.0E+01 | | 5.3E+00 | | 5.6E+00 | | 1.8E+00 | | 4.4E+00 | | 3.9E+00 | | 7.3E+00 | | 4.1E+00 |
| FEU | 3.9E-03 | 3.4E-01 | | | 7.2E-02 | | 5.3E-02 | | 5.3E-02 | | 1.4E-03 | | 7.4E-03 | | 3.5E-02 | | 6.4E-03 | | 2.9E-02 |
| PM | 5.2E-05 | 1.2E-04 | | | 8.4E-05 | | 5.9E-05 | | 6.0E-05 | | 5.8E-06 | | 1.0E-05 | | 5.8E-05 | | 1.1E-04 | | 8.6E-05 |
| IR | 1.4E+01 | 3.8E+01 | | | 3.2E+01 | | 4.5E+01 | | 4.5E+01 | | 7.0E-01 | | 1.4E+00 | | 7.4E-01 | | 8.2E+00 | | 8.8E-01 |
| HTOX_c | 4.7E-06 | 7.6E-05 | | | 4.1E-05 | | 4.8E-05 | | 4.8E-05 | | 4.2E-07 | | 4.1E-06 | | 1.7E-06 | | 2.5E-06 | | 2.3E-06 |
| ECOTOX | 1.6E+02 | 3.2E+03 | | | 1.6E+03 | | 1.3E+03 | | 1.3E+03 | | 1.9E+03 | | 2.5E+02 | | 1.8E+03 | | 2.4E+02 | | 2.8E+03 |
| HTOX_nc | 4.1E-05 | 7.9E-04 | | | 2.0E-04 | | 1.8E-04 | | 1.8E-04 | | 5.2E-06 | | 2.9E-05 | | 8.6E-06 | | 7.5E-06 | | 1.2E-05 |
| LU | 1.6E+03 | 2.3E+03 | | | 3.4E+03 | | 1.6E+03 | | 1.6E+03 | | 2.5E+03 | | 1.8E+04 | | 4.0E+04 | | 1.4E+04 | | 2.3E+04 |
| WU | 4.7E+02 | 4.3E+02 | | | 1.3E+03 | | 5.3E+02 | | 5.3E+02 | | 4.8E+02 | | 9.0E+00 | | 1.4E+01 | | 1.4E+03 | | 1.5E+01 |
| FRD | 1.5E+04 | 5.9E+03 | | | 1.3E+04 | | 7.3E+03 | | 7.2E+03 | | 3.6E+02 | | 5.3E+02 | | 3.9E+02 | | 1.0E+03 | | 5.4E+02 |
| MRD | 1.8E-04 | 2.0E-02 | | | 1.0E-02 | | 4.6E-03 | | 4.6E-03 | | 8.7E-05 | | 6.1E-05 | | 7.3E-05 | | 2.1E-04 | | 7.3E-05 |
|  |  |  | | |  | |  | |  | |  | |  | |  | |  | |  |
|  | 25-Lime, cements and other materials | | 26-Ores, slag and ash | 27-Fuels and mineral oils | | 28-Inorganic chemicals | | 29-Organic chemicals | | 31-Fertilisers | | 39-Plastics | | 44-Wood and products | | 47-Pulp of wood or other cellulosic material | | 72-Iron and steel | |
| CC | 3.4E+00 | | 1.3E+01 | 4.5E+01 | | 2.9E+02 | | 8.6E+01 | | 1.6E+02 | | 2.2E+02 | | 7.8E+00 | | 9.6E+01 | | 2.0E+02 | |
| ODP | 6.0E-12 | | 1.5E-07 | 7.0E-05 | | 1.6E-10 | | 1.9E-05 | | 2.7E-05 | | 8.6E-08 | | 9.1E-11 | | 9.9E-06 | | 5.0E-10 | |
| AC | 7.4E-02 | | 3.8E-01 | 1.2E+00 | | 2.2E-01 | | 3.8E-01 | | 9.1E-01 | | 1.2E+00 | | 4.1E-02 | | 1.3E+00 | | 9.7E-01 | |
| POF | 5.1E-02 | | 2.8E-01 | 4.6E-01 | | 1.8E-01 | | 3.1E-01 | | 3.0E-01 | | 1.2E+00 | | 3.9E-02 | | 9.8E-01 | | 6.3E-01 | |
| MEU | 1.8E-02 | | 9.9E-02 | 9.6E-02 | | 1.9E-01 | | 9.3E-02 | | 1.2E-01 | | 2.4E-01 | | 1.6E-02 | | 3.5E-01 | | 1.9E-01 | |
| TEU | 2.0E-01 | | 1.1E+00 | 1.0E+00 | | 6.2E-01 | | 1.0E+00 | | 3.1E+00 | | 2.6E+00 | | 1.7E-01 | | 3.6E+00 | | 2.0E+00 | |
| FEU | 5.5E-06 | | 1.3E-04 | 1.1E-03 | | 2.7E-04 | | 2.2E-03 | | 5.4E-03 | | 1.1E-04 | | 2.6E-05 | | 5.8E-03 | | 2.9E-04 | |
| PM | 5.7E-07 | | 8.9E-06 | 1.1E-05 | | 1.7E-06 | | 3.4E-06 | | 2.1E-05 | | 1.1E-05 | | 5.2E-07 | | 2.3E-05 | | 3.1E-05 | |
| IR | 1.7E-01 | | 4.1E-01 | 2.7E+00 | | 8.4E+00 | | 1.9E+00 | | 4.8E+00 | | 7.5E-02 | | 1.7E+00 | | 8.3E+00 | | 1.2E+00 | |
| HTOX_c | 1.3E-08 | | 1.6E-07 | 9.3E-07 | | 5.9E-08 | | 4.0E-07 | | 1.5E-06 | | 1.6E-06 | | 3.0E-08 | | 2.7E-06 | | 1.2E-06 | |
| ECOTOX | 2.6E-01 | | 1.1E+01 | 7.5E+01 | | 2.5E+00 | | 2.2E+01 | | 1.4E+02 | | 9.3E+01 | | 8.7E-01 | | 1.0E+02 | | 2.7E+01 | |
| HTOX_nc | 7.8E-08 | | 6.3E-07 | 5.2E-06 | | 6.0E-07 | | 2.8E-06 | | 1.3E-05 | | 1.5E-06 | | 3.1E-07 | | 1.1E-05 | | 5.2E-05 | |
| LU | 1.2E+01 | | 9.8E+00 | 3.3E+02 | | 1.3E+02 | | 4.9E+01 | | 2.8E+02 | | 4.3E+00 | | 6.3E+02 | | 7.5E+04 | | 1.8E+02 | |
| WU | 3.4E+00 | | 9.2E+00 | 3.3E+01 | | 3.7E+01 | | 5.2E+01 | | 4.1E+01 | | 1.4E+02 | | 1.6E+01 | | 4.1E+02 | | 4.6E+01 | |
| FRD | 4.2E+01 | | 1.6E+02 | 5.0E+03 | | 3.6E+03 | | 3.5E+03 | | 2.8E+03 | | 7.3E+03 | | 1.2E+02 | | 1.4E+03 | | 2.0E+03 | |
| MRD | 6.3E-07 | | 5.1E-06 | 1.2E-05 | | 2.3E-05 | | 6.2E-05 | | 5.6E-04 | | 5.3E-06 | | 1.7E-06 | | 2.7E-04 | | 1.6E-06 | |

Table 6: Impact intensities, expressed as impact per 100 kg of product, calculated for exports in 2010

|  | 10-Cereals | 25-Lime, cements and other materials | 26-Ores, slag and ash | 27-Fuels and mineral oils | 28-Inorganic chemicals | 29-Organic chemicals | 30-Pharmaceuticals | 31-Fertilisers | 39-Plastics | 44-Wood and products |
| --- | --- | --- | --- | --- | --- | --- | --- | --- | --- | --- |
| CC | 5.5E+01 | 8.3E+01 | 1.8E+00 | 8.4E+01 | 1.3E+01 | 1.2E+02 | 9.1E+02 | 5.8E+01 | 2.1E+02 | 1.5E+01 |
| ODP | 3.8E-06 | 2.9E-06 | 1.5E-07 | 1.2E-10 | 1.5E-06 | 8.0E-06 | 6.4E-05 | 7.8E-06 | 8.6E-08 | 2.0E-11 |
| AC | 6.1E-01 | 1.5E-01 | 3.1E-02 | 3.5E-01 | 1.7E+00 | 4.5E-01 | 4.6E+00 | 2.1E-01 | 7.8E-01 | 7.6E-02 |
| POF | 1.7E-01 | 1.4E-01 | 2.8E-02 | 2.3E-01 | 1.9E-01 | 4.2E-01 | 2.2E+00 | 1.2E-01 | 8.6E-01 | 8.9E-02 |
| MEU | 1.2E+00 | 4.6E-02 | 9.4E-03 | 9.3E-02 | 3.1E-02 | 8.5E-02 | 6.2E+00 | 3.2E-02 | 1.3E-01 | 3.4E-02 |
| TEU | 2.6E+00 | 5.4E-01 | 1.4E-01 | 6.8E-01 | 3.4E-01 | 9.4E-01 | 7.3E+00 | 3.6E-01 | 1.4E+00 | 3.7E-01 |
| FEU | 8.3E-03 | 4.6E-04 | 1.3E-04 | 1.3E-03 | 1.7E-03 | 3.4E-03 | 8.6E-02 | 2.9E-03 | 1.1E-04 | 6.5E-05 |
| PM | 6.9E-06 | 1.8E-06 | 6.1E-06 | 3.0E-06 | 1.2E-05 | 5.3E-06 | 4.6E-05 | 4.3E-06 | 7.6E-06 | 2.1E-05 |
| IR | 1.2E+00 | 2.6E+00 | 3.1E-01 | 1.6E+00 | 1.0E+00 | 1.9E+00 | 8.6E+01 | 1.8E+00 | 4.7E-02 | 8.0E-01 |
| HTOX_c | 3.1E-06 | 1.4E-07 | 8.7E-08 | 2.4E-06 | 4.8E-07 | 1.1E-06 | 2.0E-05 | 8.4E-07 | 1.5E-06 | 5.6E-08 |
| ECOTOX | 2.5E+02 | 9.0E+00 | 9.5E+00 | 5.3E+01 | 2.7E+01 | 5.9E+01 | 1.3E+03 | 5.6E+01 | 9.2E+01 | 1.3E+00 |
| HTOX_nc | 7.6E-06 | 3.8E-06 | 3.8E-07 | 9.3E-06 | 3.9E-06 | 6.9E-06 | 2.7E-05 | 6.8E-06 | 1.2E-06 | 4.6E-07 |
| LU | 1.6E+04 | 6.4E+01 | 7.4E+00 | 2.4E+03 | 1.0E+02 | 1.9E+02 | 3.0E+04 | 1.8E+02 | 3.7E+00 | 2.9E+03 |
| WU | 1.3E+01 | 1.4E+01 | 8.6E+00 | 4.3E+01 | 2.2E+02 | 8.1E+01 | 1.2E+03 | 1.6E+01 | 1.4E+02 | 7.2E+00 |
| FRD | 3.0E+02 | 3.4E+02 | 3.0E+01 | 5.1E+03 | 1.9E+02 | 5.5E+03 | 1.9E+04 | 8.6E+02 | 7.1E+03 | 1.9E+02 |
| MRD | 1.8E-04 | 1.3E-05 | 4.8E-06 | 2.4E-05 | 2.3E-04 | 3.8E-04 | 4.0E-03 | 3.6E-04 | 5.0E-06 | 4.0E-06 |
|  | 47-Pulp of wood or other cellulosic material | 48-Paper and products | 71-Precious materials | 72-Iron and steel | 73-Articles of iron or steel | 84-Machineries | 85-Electrical equipment | 87-Vehicles | 88-Aircrafts | 90-Precision instruments |
| CC | 8.2E+01 | 1.4E+02 | 3.8E+02 | 2.0E+02 | 2.8E+02 | 4.0E+02 | 1.0E+02 | 4.0E+02 | 1.3E+03 | 7.1E+02 |
| ODP | 9.9E-06 | 1.3E-05 | 2.7E-05 | 1.0E-05 | 1.4E-06 | 2.9E-05 | 5.7E-05 | 2.5E-05 | 6.0E-05 | 4.8E-05 |
| AC | 7.9E-01 | 8.5E-01 | 6.1E+00 | 8.8E-01 | 7.4E-01 | 4.1E+00 | 8.0E-01 | 3.0E+00 | 1.1E+01 | 4.1E+00 |
| POF | 6.4E-01 | 5.0E-01 | 1.5E+00 | 6.3E-01 | 4.7E-01 | 1.5E+00 | 4.0E-01 | 1.7E+00 | 3.6E+00 | 2.1E+00 |
| MEU | 2.3E-01 | 1.8E-01 | 3.8E-01 | 1.6E-01 | 1.2E-01 | 4.3E-01 | 8.4E-02 | 3.0E-01 | 1.1E+00 | 6.5E-01 |
| TEU | 2.3E+00 | 1.7E+00 | 4.3E+00 | 1.8E+00 | 1.3E+00 | 5.0E+00 | 9.2E-01 | 3.4E+00 | 1.2E+01 | 7.1E+00 |
| FEU | 5.8E-03 | 9.6E-03 | 1.6E-01 | 1.5E-02 | 3.3E-05 | 3.4E-01 | 1.9E-03 | 5.3E-02 | 1.2E-01 | 4.3E-02 |
| PM | 1.9E-05 | 1.8E-05 | 1.1E-04 | 4.4E-05 | 7.1E-06 | 1.1E-04 | 1.1E-05 | 5.4E-05 | 1.3E-04 | 3.1E-04 |
| IR | 8.3E+00 | 2.5E+01 | 3.5E+01 | 1.1E+01 | 3.7E-01 | 3.8E+01 | 4.3E+00 | 4.5E+01 | 2.2E+02 | 6.0E+01 |
| HTOX_c | 2.6E-06 | 2.4E-06 | 6.9E-05 | 5.8E-05 | 8.7E-08 | 7.6E-05 | 1.3E-06 | 4.8E-05 | 2.9E-05 | 3.2E-04 |
| ECOTOX | 1.0E+02 | 2.0E+02 | 2.4E+03 | 1.3E+03 | 5.5E+00 | 3.2E+03 | 7.1E+01 | 1.3E+03 | 2.2E+03 | 9.1E+03 |
| HTOX_nc | 1.1E-05 | 1.9E-05 | 4.0E-04 | 1.1E-04 | 4.9E-06 | 7.9E-04 | 7.0E-06 | 1.8E-04 | 3.0E-04 | 2.4E-04 |
| LU | 7.5E+04 | 3.6E+04 | 2.1E+03 | 8.0E+02 | -2.8E-04 | 2.3E+03 | 2.7E+02 | 1.6E+03 | 2.7E+03 | 4.0E+03 |
| WU | 4.1E+02 | 4.6E+02 | 4.0E+02 | 2.2E+02 | 1.9E+01 | 4.3E+02 | 7.8E+01 | 5.3E+02 | 1.7E+03 | 5.7E+02 |
| FRD | 1.3E+03 | 2.7E+03 | 5.5E+03 | 2.7E+03 | 2.7E+03 | 5.8E+03 | 5.1E+03 | 7.0E+03 | 2.1E+04 | 9.9E+03 |
| MRD | 2.7E-04 | 2.5E-04 | 8.3E-03 | 2.2E-03 | 4.4E-06 | 2.0E-02 | 3.7E-05 | 4.6E-03 | 7.4E-03 | 1.8E-02 |

1. **Contribution of substances**

Figure 1 reports the contribution of elementary flows, i.e. environmental emissions and resources, to the analysed impact categories. It refers to exports in 2010.

Figure 1: Share of substances for export. Only substances contributing to more than 3% to impact categories are reported, substances with lower contributions are grouped under the category “other flows”. CC=climate change, ODP=ozone depletion, AC=acidification, POF=photochemical ozone formation, MEU=eutrophication, marine, TEU=eutrophication, terrestrial, FEU=ecotoxicity, freshwater, PM=particulate matter, IR=ionizing radiation, HTOX_c=human toxicity, cancer, ECOTOX=ecotoxicity, HTOX_nc=human toxicity, non cancer, LU=land use, WU=water use, ,FRD=resource use, fossils, MRD=resource use, mineral and metals.

1. **Normalization and weighting factors**

Table 5 reports the list of normalization factors and weighting factors used in this study. Global normalization factors are subject to frequent updates. The one used in this study result from an update of the normalization factors published by Sala et al. (2017). Figures in blue in Table 5 refer to updated normalization factors compared to those in the source. Weighting factors are the ones developed for the EU Environmental Footprint (Sala et al., 2018).

Table 7: Global normalization factors (updated from Sala et al., 2017, figures in blue refer to updated normalization factors compared to those in the source) and weighting factors used in this study (Sala et al.2018)

| **Impact category** | **Global normalization factors** | | **Weighting factors** |
| --- | --- | --- | --- |
| Climate change | 5.55E+13 | kg CO_2_ eq | 12.9 |
| Ozone depletion | 3.33E+08 | kg CFC-11 eq | 5.58 |
| Human toxicity, non-cancer | 3.27E+06 | CTUh | 5.88 |
| Human toxicity, cancer | 2.66E+05 | CTUh | 6.8 |
| Particulate matter | 4.11E+06 | Disease incidence | 5.49 |
| Ionising radiation | 9.54E+11 | kBq U^235^ eq | 5.7 |
| Photochemical ozone formation | 2.80E+11 | kg NMVOC eq | 4.76 |
| Acidification | 3.83E+11 | molc H^+^ eq | 4.94 |
| Eutrophication, terrestrial | 1.22E+12 | molc of N eq | 2.95 |
| Eutrophication, freshwater | 1.11E+10 | kg P eq | 3.19 |
| Eutrophication, marine | 1.35E+11 | kg N eq | 2.94 |
| Ecotoxicity freshwater | 8.15E+13 | CTUe | 6.12 |
| Land use | 1.54E+16 | Pt | 9.04 |
| Water use | 7.91E+13 | m³ | 9.69 |
| Resource use, fossils | 4.48E+14 | MJ | 7.37 |
| Resource use, mineral and metals | 4.39E+08 | kg Sb eq | 6.68 |

**References**

Sala S., Crenna E., Secchi M., Pant, R., Global normalisation factors for the Environmental Footprint and Life Cycle Assessment, EUR (28984), Publications Office of the European Union, Luxembourg, 2017, ISBN 978-92-79-77213-9, doi:10 .2760/889.

Sala S., Cerutti A.K., Pant R., 2018. Development of a weighting approach for the Environmental Footprint, Publications Office of the European Union, Luxembourg, 2018, ISBN 978-92-79-68042-7, doi:10.2760/945290.
